# Comparison of simulation and video-based training for acute asthma

**DOI:** 10.1186/s12909-023-04836-7

**Published:** 2023-11-16

**Authors:** Mohamed Habib Grissa, Randa Dhaoui, Khaoula Bel Haj Ali, Adel Sekma, Maroua Toumia, Sarra Sassi, Abdel Karim Sakly, Asma Zorgati, Hajer Bouraoui, Houda Ben Soltane, Zied Mezgar, Riadh Boukef, Hamdi Boubaker, Wahid Bouida, Kaouthar Beltaief, Semir Nouira

**Affiliations:** 1 Emergency Department, Fattouma Bourguiba University Hospital, Monastir, 5000 Tunisia; 2 Research Laboratory LR12SP18, University of Monastir, Monastir, 5000 Tunisia; 3 Emergency Department, Haj Ali Soua Regional Hospital of Ksar Hellal, Ksar Hellal, 5070 Tunisia; 4 Orthopedic Department, Fattouma Bourguiba University Hospital, Monastir, 5000 Tunisia; 5 Emergency Department, Sahloul University Hospital, Sousse, 4000 Tunisia; 6 Pharmacology Department Faculty of Medicine, University of Monastir, Monastir, 5000 Tunisia; 7 Emergency Department, Farhat Hached University Hospital, Sousse, 4000 Tunisia; 8 Emergency Department and Laboratory Research (LR12SP18), Fattouma Bourguiba University Hospital, Monastir, 5000 Tunisia

**Keywords:** Acute asthma, High-fidelity simulation, Interactive video-case education

## Abstract

**Background:**

Emergency medicine is particularly well suited to simulation training. However, evidence for the efficacy of simulation-based medical training remains limited especially to manage high-risk cases such as acute asthma.

**Objective:**

The objective of our study was to compare the performance of high-fidelity simulation (HFS) and interactive video-case challenge-based training (IVC) for final-year medical students in the management of acute asthma.

**Methods:**

This was a prospective randomized controlled study conducted at the emergency department (ED) of Monastir University hospital ( Tunisia). 69 final-year medical students were randomized to HFS (*n* = 34) and IVC (*n* = 35) training on acute asthma topic. The study was conducted over a 1-week period. Efficacy of each teaching method was compared through the use of multiple-choice questionnaires (MCQ) before (pre-test), after (post-test) training and a simulation scenario test conducted 1 week later. The scenario was based on acute asthma management graded on predefined critical actions using two scores: the checklist clinical score (range 0 to 30), and the team skills score (range 0 to 16). Student satisfaction was also evaluated with the Likert 5 points scale. Two years after the post-test, both groups underwent a third MCQ testing to assess sustainability of knowledge.

**Results:**

There were no differences in age between groups. There was no statistically significant difference between the HFS and IVC groups pre-test scores (*p* = 0.07). Both groups demonstrated improvement in MCQ post-test from baseline after training session; the HFS MCQ post-test score increased significantly more than the IVC score (*p* < 0.001). The HFS group performed better than the IVC group on the acute asthma simulation scenario (*p* < 0.001). Mean checklist clinical score and mean team skills score were significantly higher in HFS group compared to IVC group (respectively 22.9 ± 4.8 and 11.5 ± 2.5 in HFS group vs 19.1 ± 3 and 8.4 ± 3.1 in IVC group) (*p *< 0.001). After 2 years, MCQ post-test scores decreased in both groups but the decrease was lower in HFS group compared to the IVC group.

**Conclusion:**

High-fidelity simulation-based training was superior to interactive video-case challenge for teaching final year medical students,and led to more long-term knowledge retention in the management of simulated acute asthma patients.

**Trial registration:**

The study was registered at www.clinicaltrials.gov NCT02776358 on 18/05/2016.

**Supplementary Information:**

The online version contains supplementary material available at 10.1186/s12909-023-04836-7.

## Background

The acquisition of clinical skills can be problematic for medical students in a highly demanding environment such as Emergency Department (ED). At the start of their career, these future doctors, inadequately prepared, feel a lot of stress [1, 2] and their patients may not receive optimal care [3, 4]. In fact, in the emergency environment, medical students have limited opportunities for bedside teaching and this could affect their awareness and self-confidence [5]. Improving the quality and efficiency of their training is required.

Medical simulation has become a widely spread and effective method of medical education [6–9]. Studies thus far showed that the use of simulation for the training of medical students and residents is helpful to access to practical “hands-on” applications of their theoretical knowledge, and to develop their technical and non-technical skills in safe realistic environments [10] Simulations that present highly realistic performance characteristics, contexts, and scenarios are referred to as high-fidelity, while low-fidelity simulators are partial-task trainer devices and screen-based video game. Higher levels of fidelity can enhance participants’ level of engagement and acceptability of the simulated experience; this will impact the achievement of the desired learning objectives and the ability to transfer the learning to the clinical setting [11]. This method is capable of both simulating realistic patient encounters and giving real-time physiologically accurate feedback. In another hand, Simulation has become a frequently used evaluation method. It helps to assess the first three levels of learning given the ability to choose the program and select learner-specific findings, conditions, scenarios; providing standardized experiences for all trainees; and measure the outcome with reliable data.

High fidelity simulation was widely used in anesthesiology within a multitude of topics such as airway and hemodynamic management [12], per and peri-operative anaesthesia, crisis resource management (CRM) [13] and more recently ultrasound-guided regional anesthesia [14].

Moreover, high fidelity simulation High fidelity simulation has spread from anesthesiology to other disciplines including emergency medicine [15–17]. Simulation training has added the advantage of being available whenever needed and does not rely on random patient encounters for medical education [10]. Emergency medicine is uniquely suited to learning through simulation. Simulation allows medical students to manage rare and high-risk cases in a safe environment without patient risk. It is indeed proven that simulation courses improve the confidence and the performance of doctors [8, 9]. The requirements regarding knowledge should focus on the problems that are frequently encountered in the ED, for instance, protocols on acute asthma management. Acute asthma exacerbation is frequently encountered in the ED, and its early diagnosis and treatment is crucial to preventing disease complications [18]. There have been no studies that prospectively compare a standard method with high-fidelity simulation for acute asthma management training. In addition, no studies to date have evaluated or compared the long-term retention of knowledge with the two learning methods.

The objective of this prospective randomized study was to determine whether simulation training is superior to video case challenge for teaching acute asthma management to final-year medical students. The use of video case challenge as a comparison was selected, as this modality is now frequently used in our ED for undergraduate medical students.

## Methods

### Participants and setting

We conducted this prospective, randomized, non-blinded study during the academic year 2015–2016 in the ED simulation laboratory of the ED of Fattouma Bourguiba University Hospital according to the ICH-GCP guidelines (International Conference on Harmonisation-Good Clinical Practice) as well as the Declaration of Helsinki. Ethics approval was obtained from the Fattouma Bourguiba University Hospital Ethics Committee. The study population included final-year medical students rotating through a 4-week Emergency Medicine attachment as part of their final-year medicine curriculum. The sample was convenience-based. Following informed consent, each group was randomized to one of two teaching methods: high fidelity simulation-based training (HFS group) or interactive video case challenge (IVC group). The topic was the management of an acute asthma patient at the ED. Each method of this teaching session was carefully prepared to give the same key concepts to allow the student to recognize the severity of the disease and manage the patients according to the current guidelines [19]. The objectives were clinically focused and specifically designed to include elements that would be necessary to successfully care for patients. All teaching sessions were performed by the same emergency physicians seniors.

### Measurements

**Fig. 1:**
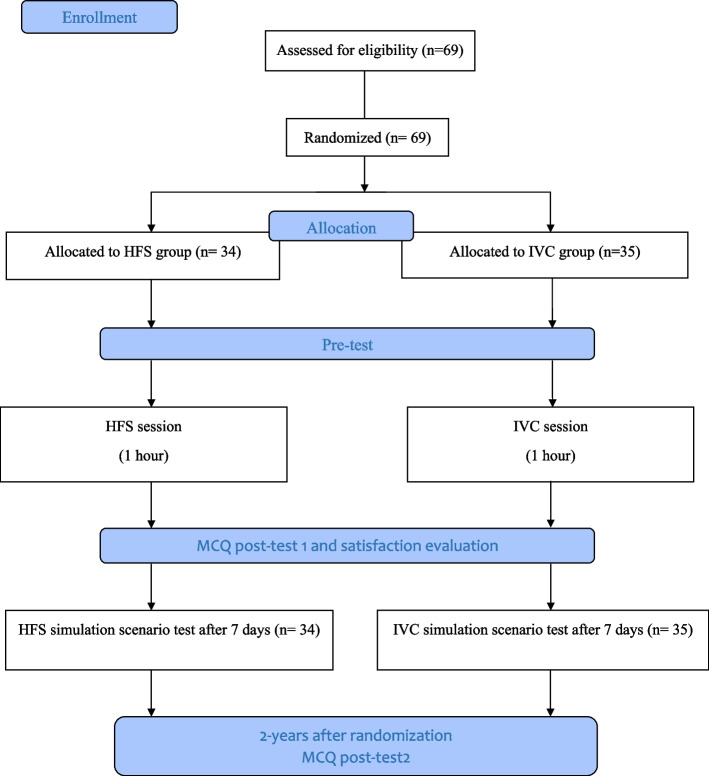
The study population flowchart. IVC teaching group: interactive video case teaching group. HSF group: high fidelity simulation-based teaching group. MCQ: multiple choice questions

Both groups underwent a baseline testing (pre-test) including 20 Multiple Choice Questions (MCQ) for 20 min about their baseline knowledge of acute asthma management. The Questions focused on anamnestic and clinical diagnostic management, differential diagnoses recognition,severity assessment and detailed therapeutic management. The MCQ test score range from 0 (minimum) to 20 (maximum). Students completed a second MCQ immediately following the teaching session (MCQ post-test 1). The results of all MCQ examinations were fed back to each participating student upon completion of this teaching session and MCQ examination. After randomization, all students received an equivalent 30 min orientation to the human patient simulator (Laerdal SimMan® full scale patient simulator; Laerdal Medical Corporation, NY), in the ED simulation lab: realistic full-body adult patient simulator SimMan®3G (Laerdal company Prod No: 212–02050 serial NR 21244154781. Made in Norway). This simulator offers a multitude of respiratory signs such as all normal and pathological audible sounds with the stethoscope in 5 anterior and 6 posterior auscultatory sites as well as cyanosis, chest expansion. It is also possible to monitor pulse, oxygen saturation, heart rate and blood pressure non-invasively. These sessionincluded an introduction and a review of the simulator features as well as the physiologic monitoring devices. Students were instructed to verbalize their thoughts, orders, and actions during the simulated patient scenario. Simulation case scenario of acute asthma was developed by the authors and reviewed by an advisory committee. The execution of the simulation scenario required two instructors, one to engage within the scenario with the students, and a second to coordinate computer driven physiological responses dependent on intervention implemented by students. IVC group, attend real video projection filmed in ED after consent of both patient and healthcare team. The students of HFS group participated in a simulation session with an acute asthma scenario including the three known steps: briefing, scenario, and debriefing. The clinical scenario used is the same viewed in the video case as well as practiced on the simulator, however on the high fidelity simulator it was developed and programmed on the mannequin manually using the integrated software (LLEAP® version 5.1.0: 2015). Both teaching sessions lasted approximately 1 h. After the teaching sessions were complete, the students underwent gimmediately the post-test. Students also rated their satisfaction level with a 5 points Likert scale framed as attitude toward simulation compared with control group: dissatisfied (1 point), fairly satisfied (2 points), neither satisfied (3 points), satisfied (4 points) and very satisfied (5 points). The study population flowchart is depicted in Fig. 1. Seven days after the two teaching procedures, all students participated in a simulation scenario test on another acute asthma case that differs from the previous training scenario. Two emergency senior physicians independently scored each student’s performance during the simulation scenario test. Each rater individually rated the video files using two rating scores. The first rating score (checklist clinical score) included 15 items related to critical actions specific to acute asthma. Individual actions were weighted by consensus. Components of the evaluation grid were history, physical examination, diagnostic of acute asthma exacerbation, severity assessment, and treatment (Supplementary file 1). The second rating score (team skills score) used the first 8 items of a previously validated behavioral rating scale, the Mayo High Performance Teamwork Scale [20] (Supplementary file 2). Each item was scored from 0 if not performed, 1 if it was imperfectly performed and 2 if it was performed correctly. The checklist score ranged from 0 to 30, and the team skills score ranged from 0 to 16. No formal inter-rater reliability calculation was performed. Each rater was chosen for his expertise as an acute care physician and crisis resource management instructor. The simulator instructor present for all simulator sessions was excluded from being a rater to preserve the integrity of the blinding process. Differences between the raters’ evaluation were resolved by consensus. The sustainability of the acquired knowledge was assessed through the completion of an MCQ test 2 years following the teaching session (MCQ post-test 2). Students were contacted either by email or private messaging to assess their ability to answer the same MCQ test with an online application for creating and distributing questionnaires and collecting data (ASKABOX ® online free version).

### End point assessment

Primary end point was the combined simulation scenario scores (checklist clinical score and team skills score). Secondary end point included improvement in MCQ test scores (MCQ post-test 1 and MCQ post-test 2) compared to pre-test MCQ and satisfaction among HFS group and IVC group. We defined the delta MCQ score (Δ-score) as the difference between MCQ post-tests and pre-test MCQ scores.

$$\mathrm\Delta-\mathrm{score}\;(\%)=\;(\mathrm{MCQ}\;\mathrm{post}-\mathrm{test}\;-\;\mathrm{MCQ}\;\mathrm{pre}-\mathrm{test})\;/\;\mathrm{MCQ}\;\mathrm{pre}-\mathrm{test}\;\;\mathrm x\;100$$

### Data analysis

Statistical analysis was performed with SPSS version 21.0. Pre- and post-simulation MCQ test scores, simulation scenario test score, and satisfaction survey responses were summarized by descriptive statistics. Data were analyzed by the Kolmogorov–Smirnov test (K-S test) to assess normality and were expressed as mean ± standard deviation or median and interquartile range (IQR). The Mann–Whitney test was used to analyze differences between the two groups in pre-simulation test scores, post-simulation test scores, simulation scenario test score, and satisfaction scales. The Wilcoxon's Sign Rank test was used to analyze the difference between the pre-test and post-test scores in each group.

## Results

Sixty-nine final year medical students were included in the study population, median age was 23 years [21, 22] and 67.7% of the participating students were female. Each group was divided into 11 subgroups of 3 to 4 students. Results of all MCQ tests are depicted in Table 1. There was not a statistically significant difference between HFS group and IVC group mean scores at pre-test MCQ (*p* = 0.07). After training session, the HFS group mean post-test 1 score was higher than the IVC score (14.5 ± 1.6 vs 13.9 ± 1.6, respectively; (*p* < 0.001). Both groups had improved significantly from their pre-test scores with Δ-score 1 in HFS group of 82.4% ± 73.7 and 47.2% ± 32.4 in IVC group (*p* < 0.001) (Table 1). On the simulation scenario test, the HFS groups mean checklist clinical score was significantly higher than mean IVC group’s (22.9 ± 4.8 vs 19.1 ± 3; *p* < 0.001)). In addition, the mean HFS group’s team skills score was significantly higher than mean IVC group’s score (11.5 ± 2.5 vs 8.4 ± 3.1; *p* < 0.001) Table 2. The results of the satisfaction survey were in favor of HFS teaching compared to IVC teaching (Table 2). After 2 years, the MCQ post-test 2 scores were lower in both groups than MCQ post-test 1 results. Although this difference was lower in the HFS group -1.5 [-7,75–0.3] vs -3.5 [-9.2- -1.25], with no statistically significant difference. These MCQ post-test 2 scores were both statistically improved compared with pre-test scores: Δ-score 2 was significantly higher in HFS group than the IVC group (43.7% [11.4- 75] vs 15.5% [1.3- 39.2]) (*p* = 0.017).

**Table 1:** Multiple Choice Questions (MCQ) test results

	IVC group	HFS group	*P*
MCQ Pre-test score, mean (/20)	8.8 (± 2.5)	9.8 (± 1.8)	0.065
MCQ Post-test 1 score, mean (/20)	13.9 (± 1.6)	14.5 (± 1.6)	0.160
Delta-score 1, mean (%)	47.2 ± 32.4	82.4 ± 73.7	0.012

T- Student test was used

Δ-score 1 (%) = (MCQ post-test 1 – MCQ pre-test) / MCQ pre-test × 100

*IVC group* interactive video case teaching group, *HSF group* high fidelity simulation-based teaching group

**Table 2:** Check list, team skills and satisfaction scores

	IVC group	HFS group	*P*
Checklist clinical score, mean (SD)	19.1 (3)	22.9 (4.8)	< 0.001
Team skills score, mean (SD)	8.4 (3.1)	11.5 (2.5)	< .0001
Satisfaction score, median [IQR]	3.8 [-7.75–0.3]	4 [-9.2- -0.25]	0.05

T- Student test was used

*IVC group* interactive video case teaching group, *HSF group* high fidelity simulation-based teaching group, *SD* standard deviation, *IQR* interquartile range

## Discussion

This study showed that teaching the management of acute asthma patients by simulation is superior than teaching by interactive video case. This superiority was illustrated by higher improvement of MCQ scores and higher scores of clinical skills and teamwork among medical students during the execution of HF simulation scenario. In addition, our study demonstrated an improved knowledge retention among simulation-based education compared to IVC teaching with a follow-up of 2 years. The student satisfaction was also better among simulation group.

Emergency medicine poses challenges for the education of medical students who often had limited opportunities for bedside teaching during the management of vulnerable and high risk patients with the large numbers of students. Although simulation training is a good alternative, the studies examining its use are limited in the setting of acute care [13, 14, 23, 24]. The use of simulation in emergency medicine has expanded since 1990s [21, 22, 25, 26]. A systematic review of emergency medicine training has demonstrated that technology-enhanced simulation is more beneficial than traditional training [27]. Thereby, HFS was shown effective in a variety of simulated scenarios concerning urgent condition such as airway management [12], trauma management [26], and critical care management [28, 29]. A study by Steadman et al. [30] randomized fourth-year medical students to receive a problem-based learning or simulation-based teaching training intervention for the management of acute dyspnea. The results showed that the group receiving the simulation intervention performed significantly better with a greater improvement in scores from baseline than the problem-based learning group. A recent metanalysis had also evaluated the use of HF simulation in ALS training; pooled data from the RCTs demonstrated a benefit in improvement of knowledge and skill performance for HF simulation when compared with low fidelity simulation and traditional training with also greater benefit in knowledge with HF simulation compared with traditional training at the course conclusion [31]. Our results are similar to those observed in a randomized study conducted by Schroedl et al., which showed that simulator trained residents scored significantly higher on the bedside skills assessment compared with traditionally trained residents (82.5% ± 10 vs 74.8% ± 14). Another study by Ruesseler et al. evaluated the use of studied using simulation training in medical emergencies found superior performance among simulation students compared to controls [32]. Simulator-trained residents were highly satisfied with the simulation curriculum [33]. The evaluation on simulator showed that HFS is very suitable for teaching team work management as assessed by the "Mayo high performance teamwork scale". Indeed, simulation significantly increases self-confidence and the acquisition of soft skills such as communication, team interaction and leadership [17, 34]. The scenario in our study focused on the management of severe acute asthma. Asthma exacerbation is frequently encountered in respiratory medicine clinic and EDs. Failure to recognize the signs of patient deterioration on time could lead to a fatal outcome. Despite the undeniable improvement in the therapeutic regimens, asthma continues to be associated with high morbidity and mortality rates. There is evidence that the still high mortality of this disease is correlated with poor management by the health care team: prescribing errors, poor control of high-risk patients, non-compliance with recommendations, and poor management of asthma crisis situations [35]. Therefore, familiarity with the identification and management of asthma exacerbation immediately is mandatory for medical students. Most studies to date have been limited to the immediate benefits and short-term skill retention of simulation. Our study is one of the few that has followed a cohort of future physicians over such a long period of time (two years). Previous studies have shown that HF simulation did not significantly improve long-term retention of resuscitation knowledge [36–38]. It is known that knowledge and skills deteriorate at 3 months after training course without ongoing practice [39], this might result from the quality of the content, the limited duration of the training and spaced practice sessions. A study by Wayne et al. assessed the value of using simulation technology and deliberate practice showed statistically significant improvements in education outcomes, including compliance with standard ACLS protocols as well as retention of skills and knowledge after 14 months [40, 41]. Results from the two-year test showed a decline in scores after two years, as well as a decline in relative progress. However, the decrease in the IVC group is greater than that observed in the HFS group. These findings confirm the fact that simulation learning is probably more durable than the traditional learning method.

Some limitations could be discussed regarding our study. First, although the students were randomized to two groups that had equal scores on the written pretest, some unrecognized differences may still exist and could influence our results especially with regard to the relatively low sample size. Second, the choice of the IVC as the reference method in our study could be questioned as it is not a usual reference teaching method. We chose this method because it is often used as a teaching technique in our routine practice. Previous studies have revealed that students tend to prefer video cases since they perceive video modality as motivating [42], and stimulating [43]. Moreover, in one study, it was shown that video-based learning was as performant as simulation to teach a number of medical emergencies [44]. Third, objective structured clinical examination (OSCE) is the gold standard assessment method of clinical skills that assess many different qualitative aspects such as efficiency and the students’ skill performance with high reliability. But we did not use this tool in our study. Instead, MCQ were used to assess the participants’ knowledge and skill retention. Of note, MCQ are a widely used method to measure simple and complex intended learning outcomes [45]. Although better satisfaction regarding simulation training was previously demonstrated [46], it should be highlighted that it does not predict the students baseline level of clinical performance, and therefore instructors should not rely solely on students' perceptions to reflect their actual level of learning. Finally, transfer of human factor skills from simulation-based training to clinical practice is essential; however, there is limited evidence supporting the impact of simulation on patient outcomes or cost-effectiveness of training programs.

## Conclusion

This study showed that high-fidelity simulation-based training of acute asthma management is more performant compared with interactive video case teaching and showed better long term knowledge retention with more student satisfaction. More research is required to increase knowledge about the transfer of competencies to daily clinical practice.

### Supplementary Information

**Additional file 1. **

## Data Availability

Data will not be shared because we did not obtain participant consent for data sharing. The datasets generated and analyzed during the current study could be requested from the corresponding author on reasonable request.
