# Supplementary material for: Comparison of simulation and video-based training for acute asthma
Source: BMC Med Educ. 2023 Nov 16;23:873. doi: 10.1186/s12909-023-04836-7 (PMC10655321; doi:10.1186/s12909-023-04836-7)
Supplement: Supplementary file 1 — Additional file 1. [file 12909_2023_4836_MOESM1_ESM.docx]

**Table 1.** The checklist clinical score

| **Objectives** |  |
| --- | --- |
| 1-Reception and identification of the patient |  |
| 2-Early diagnosis of acute respiratory distress syndrome |  |
| 3-Monitor vital constants of the patient quickly |  |
| 4-Appropriate oxygen supplementation |  |
| 5-Relevant and targeted anamneses |  |
| 6-Assess exacerbation severity with anamnese |  |
| 7-Quality of the physical examination |  |
| 8-Early and accurate diagnosis of asthma exacerbation |  |
| 9-Peak-flow measurement |  |
| 10-Fischl score calculated |  |
| 11-Beta2 mimetics and parasympatholytics nebuliser administred |  |
| 12-Systemic corticosteroids administred |  |
| 13-Intravenous magnesium sulfate administration |  |
| 14-Patient orientation |  |
| 15-Patient informed, closely and frequently monitored |  |
| **TOTAL** | …./30 |

**Table 2.** The team skills score

| **Objectives** |  |
| --- | --- |
| 1-A leader is clearly recognized by all team members |  |
| 2-The team leader assures maintenance of an appropriate balance between command authority and team member participation  3-Each team member demonstrates a clear understanding of his or her role |  |
| 4-The team prompts each other to attend to all significant clinical indicators throughout the procedure/intervention. |  |
| 5-When team members are actively involved with the patient, they verbalize their activities aloud. |  |
| 6-Team members repeat back or paraphrase instructions and clarifications to indicate that they heard them correctly. |  |
| 7-Team members refer to established protocols and checklists for the procedure/intervention (Peak-flow, Fishl score) |  |
| 8-All members of the team are appropriately involved and participate in the activity. |  |
| ***TOTAL*** | ..../16 |
